# Tailoring Ge Nanocrystals via Ag-Catalyzed Chemical Vapor Deposition to Enhance the Performance of Non-Volatile Memory

**DOI:** 10.3390/nano16020146

**Published:** 2026-01-22

**Authors:** Chucai Guo, Qingwei Zhou, Biyuan Zheng, Hansheng Li, Fan Wu, Dan Chen, Fang Luo, Zhihong Zhu

**Affiliations:** 1 College of Advanced Interdisciplinary Studies & Hunan Provincial Key Laboratory of Novel NanoOptoelectronic Information Materials and Devices, National University of Defense Technology, Changsha 410073, China; gcc_1981@163.com (C.G.); biyuan_zheng2024@nudt.edu.cn (B.Z.); lhs@nudt.edu.cn (H.L.); 13808490405@139.com (F.W.); danchen56@outlook.com (D.C.); luofang2013@163.com (F.L.); zzhwcx@163.com (Z.Z.); 2 Nanhu Laser Laboratory, National University of Defense Technology, Changsha 410073, China

**Keywords:** Ge nanocrystals, catalytic growth, chemical vapor deposition, non-volatile memory

## Abstract

With the rapid advancement in portable electronics, artificial intelligence, and the Internet of Things, there is an escalating demand for high-density, low-voltage non-volatile memory (NVM) technologies. Germanium (Ge) nanocrystals (NCs) have emerged as a promising candidate for NVM applications; however, traditional synthesis methodologies suffer from limitations in achieving precise control over the size and density of these nanocrystals, which exert a significant influence on device performance. This study presents an innovative Ag-catalyzed chemical vapor deposition (CVD) methodology for the synthesis of Ge NCs with precisely controllable size and density on SiO_2_/Si substrates, tailored for NVM applications. Scanning electron microscopy characterization confirms the successful growth of faceted Ge NCs. Electrical characterization of the fabricated devices reveals that Ge NCs grown at temperatures ranging from 700 to 1000 °C exhibit memory windows spanning from 3.0 to 6.8 V under a ±6 V bias. Notably, the device synthesized at 900 °C demonstrates an exceptional memory window of 7.0 V under a ±8 V bias. Furthermore, the Ge NC-based NVM devices exhibit excellent charge retention characteristics. Specifically, for the device with Ge NCs grown at 700 °C, the time required to retain charge from 100% to 95% of its initial value exceeds 10 years, demonstrating long-term stable charge storage capability. These findings underscore the significant potential of this approach for the development of high-performance NVM technologies.

## 1. Introduction

Driven by the widespread proliferation and expanding applications of portable electronic products, wearable technologies, and recent advancements in artificial intelligence (AI) and Internet of Things (IoT)-related technologies, the demand for high-density, low-voltage non-volatile memory (NVM) devices has been further elevated [1,2,3,4,5]. Nanocrystals (NCs) embedded in the gate insulator of a metal–insulator–semiconductor (MIS) structure serve as the basic storage element for next-generation NVM devices with lower program/erase voltage, shorter program/erase times, and better endurance compared with conventional NVM using a poly-Si layer as a floating gate [6]. For NC-based floating gate applications, semiconductor, metal, or high-k dielectric NCs (e.g., Si, Ge, Ag, Pt, HfO_2_, or ZrO_2_) embedded in wide-bandgap dielectric layers have been extensively investigated [7,8,9,10,11,12,13]. Among these, Si- and Ge NC-based NVMs have received particular attention due to their full compatibility with the existing semiconductor industry. Si NC-based NVM was first proposed by Tiwari et al. as a replacement for the conventional floating gate in NVM [14]. Subsequent studies have demonstrated that Ge NCs are more promising for NVM applications than Si NCs because of their higher dielectric constant (~16.0) and smaller band gap (~0.6 eV) [6]. Additionally, Ge NCs have a strong coupling with the conduction channel, which can achieve a large memory window under low program/erase voltages [15]. Furthermore, in Ge NC-based NVM, the injected charges are mainly stored on the deep trapping centers, which facilitates long-term charge retention [16].

In NC-based memory devices, information is stored in NCs via charge injection or removal; thus, the size, distribution, and density of NCs, and the thickness of the dielectric layer, exert a significant influence on device performance [6]. Recently, Ge NCs have been predominantly synthesized through phase separation from Ge-supersaturated solid solutions of high-k dielectrics [17]. By definition, these supersaturated solid solutions contain a higher concentration of dissolved Ge than the solvent can typically accommodate under normal conditions. The most common methods for fabricating such Ge-supersaturated high-k dielectric solid solutions include magnetron sputtering (MS), chemical vapor deposition (CVD), Ge-ion implantation, molecular beam epitaxy (MBE), and pulsed laser deposition (PLD) [18,19,20,21,22,23,24]. Subsequent annealing can activate phase separation, leading to the formation of Ge NCs within the high-k dielectric matrix. Notably, for memory device applications, precise control over both the thickness of the tunnel oxide beneath the NC layer and the density/size of Ge NCs is critical. However, the aforementioned conventional methods often fail to achieve accurate regulation of Ge NCs size, position, distribution, and density—parameters that are essential for optimizing charge trapping density in NVM devices.

Herein, we report a novel strategy for the direct growth of Ge NCs with precisely controlled size and density on oxidized Si substrates via CVD assisted by Ag catalysts, tailored for Ge NC-based NVM applications. Specifically, the native SiO_2_ layer on the Si substrate serves as the tunnel oxide, while a thin high-k dielectric Al_2_O_3_ layer—deposited via atomic layer deposition (ALD)—functions as the control oxide. This well-engineered architecture enables accurate control over the thickness of both the tunnel and control oxide layers, and independent regulation of Ge NC density and size. Scanning electron microscopy (SEM) characterization confirms the successful controlled growth of faceted Ge NCs with tunable sizes and densities. Through this control, the charge trapping density of the resulting NVM devices can be effectively modulated. For devices fabricated using Ge NCs grown at 700 °C, 800 °C, 900 °C, and 1000 °C, the memory windows under a ±6 V bias are 3.0 V, 3.1 V, 5.0 V, and 6.8 V, respectively. Importantly, the device with Ge NCs grown at 900 °C exhibits a remarkable maximum memory window of 7.0 V when the bias voltage is swept from −8 V to 8 V and back. The time required to retain charge from 100% to 95% of its initial value exceeds 10 years for the device with Ge NCs grown at 700 °C.

## 2. Materials and Methods

*Synthesis of Ge NCs on the SiO_2_/Si substrate:* The low p-doped Si(100) wafers (ZhongNuo Advanced Material Technology Co., Ltd., Beijing, China) with 1–10 Ωcm resistivity were prepared by a standard two-step wet chemical cleaning with the native SiO_2_ remaining. Ag catalysts (ZhongNuo Advanced Material Technology Co., Ltd., Beijing, China) with 3 nm thickness were deposited on the SiO_2_/Si substrate by thermal evaporation process at a rate of 0.1 Å/s with a background vacuum of 5 × 10^−4^ Pa. After that, the substrate was loaded into a CVD (EasyTube 3000, First nano, San Jose, CA, USA) tube, and the Ge NCs were synthesized by the CVD process with a mass flow of 20 sccm diluted Germane (5% GeH_4_ in H_2_, Kodi, Foshan, China) and a pressure of 1 Torr. The growth temperature was 700, 800, 900, and 1000 °C, respectively. And the growth time of all the samples was 30 min.

*Fabrication of memory device:* After the formation of the Ge NCs, the samples were transferred from the CVD system to a homemade ALD system to deposit the thin Al_2_O_3_ capping layer (20 nm). A total of 70 nm Al (ZhongNuo Advanced Material Technology Co., Ltd., Beijing, China) was thermally deposited as the top electrodes using a metal shadow mask. To ensure a stable and low-resistance back contact with the Si substrate, a silicon wafer cutter was used to scratch the backside of the Si substrate to remove the native oxide layer and expose fresh silicon. Then the scratched area was coated with silver paste (conductive adhesive) and bonded to a conductive metal substrate to serve as the back electrode. Finally, the sample was placed on a hot stage and dried at 120 °C for 30 min to ensure full adhesion between the silver paste, Si substrate, and metal substrate.

*Characterization and measurement of memory device:* The morphology of the nanostructures was characterized by SEM (ZEISS-LEO 1450, Carl Zeiss AG, Jena, Germany) and high-resolution transmission electron microscopy (HRTEM) (JEM-2100, JEOL Ltd., Tokyo, Japan). The electrical properties were measured by a precision LCR meter (Agilent E4980A, Agilent Technologies, Inc., Santa Clara, CA, USA) with a sweep rate of 0.1 V/s.

## 3. Results

**Figure 1:**
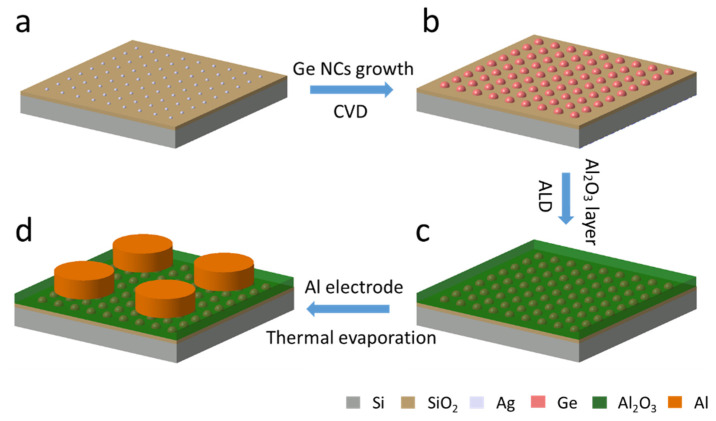
Fabrication process of p-Si/SiO_2_/Ge NC/Al_2_O_3_/Al memory structure. (**a**) Ag catalysts were deposited on the SiO_2_/Si substrates via thermal evaporation; (**b**) Ge NCs were synthesized by an Ag-catalyzed CVD process; (**c**) A thin Al_2_O_3_ capping layer was deposited via a home-built ALD system; (**d**) Al top electrodes were thermally evaporated using a metal shadow mask.

The fabrication process of the Ge NC-based NVM devices is illustrated in Figure 1, with a device structure of p-Si/SiO_2_/Ge NCs/Al_2_O_3_/Al. Specifically, the Ag catalysts were first deposited on the SiO_2_/Si substrates via thermal evaporation (Figure 1a). Subsequently, the substrates with the Ag catalysts were loaded into a CVD tube furnace, and the Ge NCs were synthesized by an Ag-catalyzed CVD process at different growth temperatures (Figure 1b). After the Ge NC formation, the samples were transferred from the CVD system to a home-built ALD system for depositing a thin Al_2_O_3_ capping layer (Figure 1c). Finally, 70 nm thick Al top electrodes were thermally evaporated using a metal shadow mask (Figure 1d). Control samples of p-Si/SiO_2_/Al_2_O_3_/Al and p-Si/SiO_2_/Ag/Al_2_O_3_/Al were deposited with the same conditions but without any Ge NCs in the intermediate layer, as shown in Figure S1.

**Figure 2:**
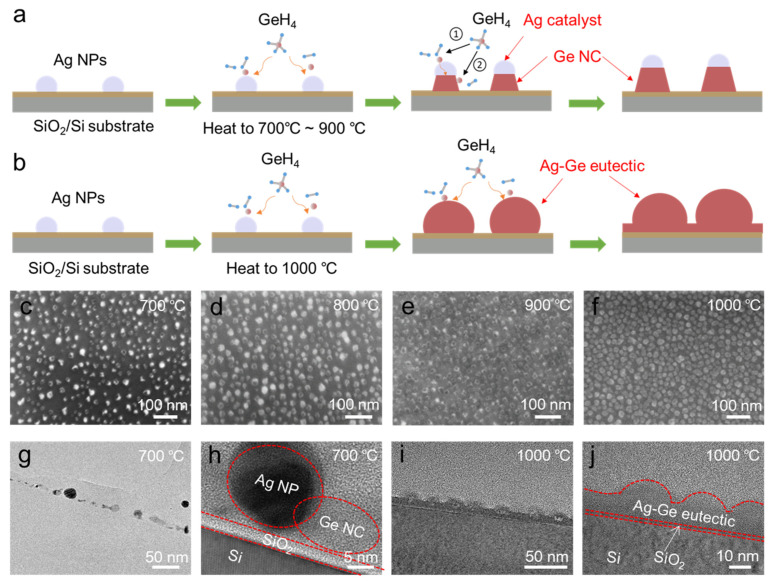
Schematic diagrams of Ag-catalyzed Ge NCs growth with different temperatures: (**a**) 700~900 °C and (**b**) 1000 °C, numbers 1, 2 in subfigure a represent the axially growing process and the radially growing process, respectively. SEM images of Ge NCs with different growth temperatures: (**c**) 700 °C, (**d**) 800 °C, (**e**) 900 °C, and (**f**) 1000 °C. TEM images of Ge NCs for (**g**,**h**) 700 °C and (**i**,**j**) 1000 °C.

The morphology of Ge NCs exerts a significant influence on the performance of Ge NC-based devices, as device information is stored in NCs via charge injection or extraction. In the temperature range of 700~900 °C, the Ag-catalyzed growth of Ge NCs follows a vapor–liquid–solid (VLS) mechanism: gaseous GeH_4_ undergoes thermal cracking to generate Ge atoms, which dissolve in Ag catalysts to form liquid Ag-Ge alloys. Subsequently, Ge atoms continuously crystallize at the catalyst interface and grow axially via catalysis; meanwhile, Ge atoms derived from GeH_4_ cracking also undergo radial non-catalytic growth along the radial direction on the side surfaces of the already grown Ge NCs. The combined effect of these two growth modes results in the formation of Ge NCs with excellent crystallinity (Figure 2a). When the growth temperature is increased to 1000 °C, Ag and Ge form an Ag-Ge eutectic phase, and Ag-Ge eutectic NCs are first grown. During the cooling process, due to the decreased solubility of Ge in the eutectic phase, Ge precipitates from the Ag-Ge eutectic, leading to slightly reduced crystallinity of the resulting Ge NCs (Figure 2b). Additionally, a higher growth temperature accelerates the growth rate of Ge NCs, thereby increasing their size and distribution density. Figure 2c–f show the SEM images of the Ag-catalyzed Ge NCs grown at different temperatures (700 °C, 800 °C, 900 °C, and 1000 °C) with approximate average sizes of 11 nm, 13 nm, 16 nm, and 19 nm. It can be observed that Ge NCs grown via the VLS mechanism at 700~900 °C exhibit a faceted morphology with the Ag catalysts attached to their tops. At 1000 °C, the faceted feature of Ge NCs becomes less pronounced, accompanied by a denser distribution and larger size. Figure 2g,h present the TEM and HRTEM images of Ge NCs grown at 700 °C, respectively. The Ag nanoparticles and Ge NCs are clearly visible, and the distinct lattice fringes confirm the excellent crystallinity of Ge NCs. The Raman spectrum of the synthesized Ge NCs at 700 °C demonstrates a sharp Ge–Ge vibration peak at 299 cm^−1^ with a FWHM of 5 cm^−1^ (Figure S2), which is consistent with the Raman characteristics of Ge NCs in the previous report [25,26], confirming good crystallization of the Ge NCs grown at 700 °C. In contrast, the Ge NCs grown at 1000 °C (Figure 2i,j) show relatively poor crystallinity.

**Figure 3:**
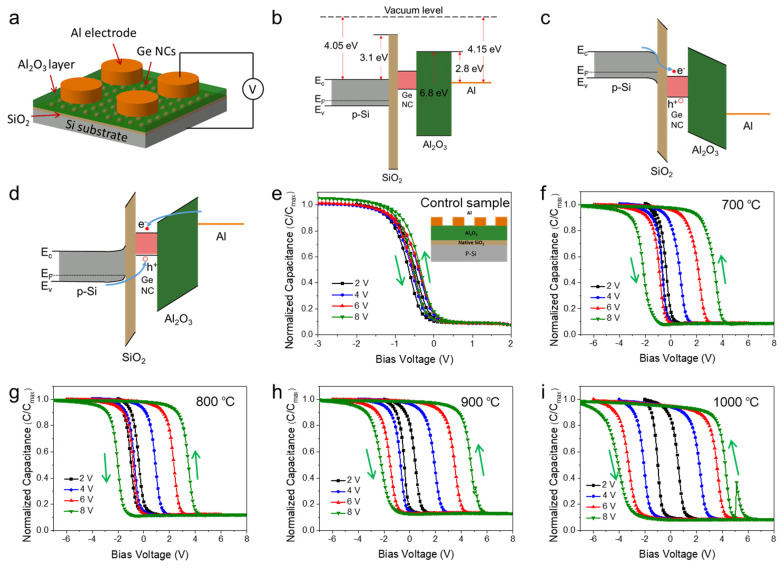
Schematic energy band diagrams of (**a**) p-Si/SiO_2_/Ge NC/Al_2_O_3_/Al memory structure at (**b**) flatband condition, and under (**c**) program and (**d**) erase modes. High-frequency (1 MHz) C-V hysteresis behavior of (**e**) the control sample and the MIS structures of Ge NCs with different growth temperatures: (**f**) 700 °C, (**g**) 800 °C, (**h**) 900 °C, (**i**) 1000 °C.

For the elucidation of the charging and discharging mechanisms, the energy band diagram of the fabricated MIS device has been drawn using the reported values of band-gap and band offset [21,27]. Figure 3a–d show the schematic device structure and energy band alignment of the p-Si/SiO_2_/Ge NCs/Al_2_O_3_/Al memory capacitor. Under the flatband condition, the memory capacitor has a potential well with a barrier height of ~2.8 eV, as shown in Figure 3b. When applying a positive bias (program mode), as shown in Figure 3c, the electrons in the channel will accumulate at the Si/SiO_2_ interface, and when the electric field is high enough, some electrons may directly tunnel through the thin SiO_2_ tunnel layer and be trapped by the Ge NCs, resulting in the right shift in the capacitance–voltage (C-V) curve. On the contrary, as shown in Figure 3d, when applying a negative bias (erase mode), the electrons stored in the Ge NCs will tunnel back to the channel, resulting in the left shift in the C-V curve. Therefore, when sweeping from negative bias to positive bias, and then reversing it, the round-trip tunneling of electrons between the channel and the Ge NCs results in the counterclockwise C-V hysteresis.

The charge storage characteristics of the Ge NCs memory devices have been studied through the C-V characteristics of MIS capacitors by sweeping the bias voltage from accumulation to depletion and back to accumulation. Figure 3e–i shows the high-frequency (1 MHz) normalized C-V hysteresis behavior of the control sample (without Ge NCs) and the MIS capacitors at various sweeping voltage ranges for the Ge NCs with different growth temperatures. The control sample of p-Si/SiO_2_/Al_2_O_3_/Al without Ag nanoparticles (NPs) and Ge NCs exhibits almost no hysteresis behavior (Figure 3e), confirming that SiO_2_/Al_2_O_3_ bulk defects or interface states contribute negligible charge trapping. In contrast, a distinct hysteresis behavior is observed in the samples containing Ge NCs (Figure 3f–i). Furthermore, in the Ge NC-based devices, it can be seen that negative bias voltages shift the C-V characteristics toward the left (corresponding to hole trapping), while positive bias voltages shift the C-V characteristics toward the right (corresponding to hole detrapping or electron trapping). The higher shift to the right than to the left indicates that the number of stored electrons is much higher than the number of stored holes [28]. Charge injection occurs from the tunneling oxide, as revealed by the counterclockwise hysteresis loop. The width of the hysteresis loop (also called memory window) widens as the sweeping voltage range becomes larger for all the NVM devices with different Ge NC growth temperatures from 700 to 1000 °C (Figure 3f–i). Concurrently, with the elevation in growth temperature, the distribution density of the Ge nanocrystals increases, enabling the device to store more charges and thus leading to an expanded memory window. Under a sweeping voltage from −8 V to 8 V, the memory window of the NVM device with Ge NCs grown at 900 °C is approximately 7.0 V (Figure 3h), which is higher than the memory windows of other Ge NC-based MIS devices reported in the literature under the same bias voltage. Detailed comparative data are presented in Table 1. The control sample of p-Si/SiO_2_/Ag NP/Al_2_O_3_/Al with only Ag NPs exhibits an extremely narrow memory window (less than 0.5 V) under the same bias-sweeping conditions used for the Ge NC-based devices (Figure S3), indicating that residual Ag NPs contribute minimally to the charge trapping and memory effect of our devices. The dominant memory performance is thus attributed to the Ge NCs. Furthermore, the memory window of the device grown at 1000 °C has achieved a maximum value of 8.3 V under a ±8 V bias due to both the Ge NCs and the defect-assisted trapping. Moreover, A sudden drop of capacitance is observed at about 5.2 V under a ±8 V bias in the C-V hysteresis in Figure 3h,i. The exact mechanism is under investigation. We ruled out instrumental error and contact resistance fluctuation by supplementing C-V measurements (Figure S4) across a broader bias range (±2 V to ±12 V) for all Ge NC devices (grown at 700 °C, 800 °C, 900 °C, and 1000 °C). Plausible causes may be interface and defect state trapping/detrapping. High-temperature CVD (1000 °C) may introduce more defects in the Ge NC and its interface compared to lower growth temperatures. When the bias reaches ~5.2 V, the strong electric field could activate these interface defects, causing a transient capture/release of carriers that disrupts the continuously changing capacitance.

**Figure 4:**
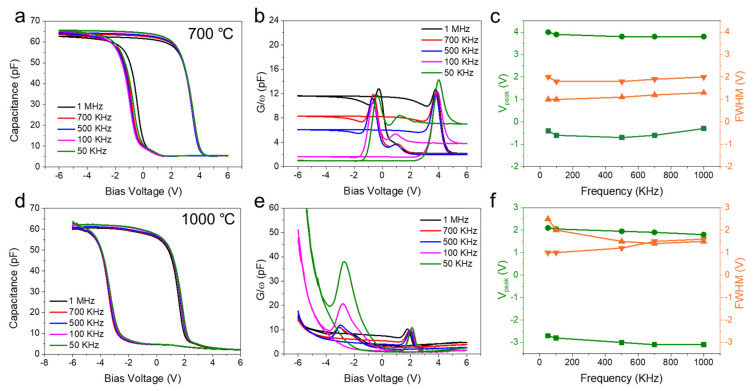
Room-temperature frequency-dependent C-V, G-V characteristics and corresponding conductance peak position and width for Ge NCs with different growth temperatures: (**a**–**c**) 700 °C and (**d**–**f**) 1000 °C. The green lines and orange lines in subfigures (**c**) and (**f**) represent the conductance peak position and the FWHM in the G-V characteristics, respectively.

The origin of the C-V hysteresis may be attributed to either charge storage in Ge NCs or interface traps in the surrounding oxide [18]. To gain deeper insights into the C-V measurement results, frequency-dependent C-V and conductance–voltage (G-V) measurements were performed on Ge NCs grown at different temperatures, with a sweeping voltage of 6 V and a frequency range of 50 Hz to 1 MHz (Figure 4). Figure 4a shows the frequency-dependent C-V characteristics of the Ge NC-based NVM device grown at 700 °C. It can be observed that the memory window does not change significantly with frequency, indicating that the main contribution to the memory effect stems from Ge NCs rather than interface states [10]. Furthermore, in the frequency range of 50 kHz to 1 MHz, the full-width-at-half-maxima (FWHM) of the conductance peak in the G-V characteristics remains nearly constant (Figure 4b,c). This phenomenon suggests that the hysteresis and the conductance peak share a common physical origin [33], with the contribution of interface trap states being deemed negligible. However, the G-V measurements revealed a slight yet discernible shift in the position of the conductance peak as a function of frequency. This behavior can be explained by the direct correlation between conductance and energy dissipation during the carrier capture and emission processes mediated by Ge NCs [34]. An identical trend was also observed in devices fabricated using Ge NCs grown at 800 °C and 900 °C (Figure S5). This consistency confirms that the origin of C-V hysteresis in these devices is also associated with charge storage within Ge NCs. Notably, for a specific frequency, the device grown at 1000 °C (Figure 4d–f). exhibits the highest conductance peak intensity, which may be due to the high density of NCs in the sample, which prevents an instantaneous response to the AC signal [35]. The carrier capture or emission processes in Ge NCs occur via tunneling through the dielectric layer to the conduction band of Ge, followed by trapping at the ground-state energy levels of the Ge NCs [16]. Consequently, the response time of Ge NC-based devices is expected to be substantially longer than that of a pure dielectric layer, where carrier capture and emission processes are exclusively mediated by interface trap states.

**Figure 5:**
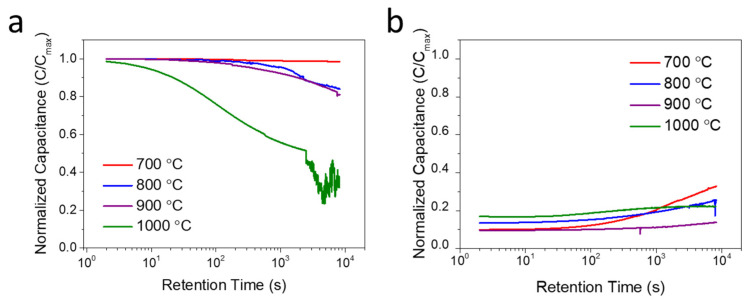
Retention characteristics of the Ge NC-based NVM under (**a**) program and (**b**) erase mode.

To evaluate the charge retention capability of Ge NC-based NVM devices with different Ge NC growth temperatures, capacitance–time (C-t) measurements were performed under zero gate bias. The C-t variation curves of the devices with Ge NCs grown at different temperatures are depicted in Figure 5. First, the Ge NCs devices were charged at a bias of +6 V for 10 s to simulate the program mode. After this charging process, the transient capacitance was measured under zero gate bias (Figure 5a). The results show that the devices with Ge NCs grown at 700–900 °C exhibit superior charge retention performance. Specifically, curve extrapolation reveals that the time required for the charge of the 700 °C sample to decay from 100% to 95% of its initial value exceeds 10 years. In contrast, the charge of the 1000 °C sample decays rapidly. It drops to less than 30% of its initial value after 10,000 s. It is found that devices with lower Ge NC growth temperatures possess better charge retention capability. This phenomenon may be attributed to the smaller size of Ge NCs formed at lower temperatures. Such a smaller size induces a stronger Coulomb blockade effect. Furthermore, higher growth temperatures lead to more defects in the Ge NC and its interface. Although high-density defects in NCs enable memory devices to store more charges, the charges stored in NCs are more prone to leakage through the surrounding oxide in high-density NC systems. Additionally, the erase state of the devices was also measured. This erase state was achieved by applying a bias of −6 V for 10 s (Figure 5b). The results demonstrate that devices with different Ge NC growth temperatures all exhibit excellent retention characteristics under the erase mode.

## 4. Conclusions

In summary, this work addresses the challenge of limited morphological control over Ge NCs in NVM applications through an Ag-catalyzed CVD approach. By precisely tuning growth parameters, this methodology enables the synthesis of faceted Ge NCs with controllable size and density, surpassing the capabilities of traditional fabrication techniques. The proposed NVM structure, consisting of a p-Si/SiO_2_/Ge NCs/Al_2_O_3_/Al stack, demonstrates remarkable performance characteristics. The memory window exhibits a positive correlation with growth temperature, achieving an exceptional value of 7.0 V under a ±8 V bias at 900 °C. Additionally, the fabricated devices show good charge retention, with less than 5% charge loss after a 10-year period for the device grown at 700 °C. These findings represent a significant advancement in Ge NCs synthesis, providing a robust foundation for the development of high-performance NVM technologies.

## Figures and Tables

**Table 1:** Comparison among the MIS memory devices based on Ge NCs.

Structure	Method	Thickness (nm)	Operating Voltage (V)	Memory Window (V)	References
SiO_2_/Ge/Al_2_O_3_	Ag-catalyzed Ge CVD	2/10/20	−8 to +8	7.0	This work
(SiO_2_/HfO_2_)/Ge/HfO_2_	Ge-HfO_2_ MS	(2.5/8)/7/22	−4 to +5	1.1	Ref. [29]
(SiO_2_/Al_2_O_3_)/Ge/Al_2_O_3_	Ge MS	(3/4)/15/10	−1 to +15	5.6	Ref. [30]
SiO_2_/Ge/SiO_2_	Ge MBE	5/1/45	−10 to +10	2.2	Ref. [5]
(SiO_2_/HfO_2_)/Ge/HfO_2_	Ge EBE	(5/5)/6/20	−4 to +4	0.8	Ref. [18]
SiO_2_/Ge/TaZrO_x_	Ge-TaZrOx MS	5/10/15	−7 to +7	5	Ref. [28]
Al_2_O_3_/Ge/Al_2_O_3_	Ge MS	4/5/15	−5 to +5	4.4	Ref. [27]
SiO_2_/Ge/SiO_2_	Ge-ion implantation	5/6/31	−4 to +4	2.1	Ref. [31]
HfAlO/Ge/HfAlO	Ge PLD	2.5/5/15	−6 to +3	3.6	Ref. [32]
SiO_2_/Ge/SiO_2_	Ge CVD	3/8.5/10	−5 to +3	3.5	Ref. [20]

## Data Availability

Data will be made available upon request.

## Supplementary Materials

The following supporting information can be downloaded at: https://www.mdpi.com/article/10.3390/nano16020146/s1, Figure S1: Preparation flowchart of control samples (p-Si/SiO_2_/Al_2_O_3_/Al and p-Si/SiO_2_/Ag/Al_2_O_3_/Al); Figure S2: The Raman spectrum of the synthesized Ge NCs at 700 °C; Figure S3: High frequency (1 MHz) C-V hysteresis behavior of the control sample (p-Si/SiO_2_/Ag NPs/Al_2_O_3_/Al); Figure S4. High frequency (1 MHz) C-V hysteresis behavior of (e) the control sample and the MIS structures of Ge NCs with different growth temperature (a) 700 °C, (b) 800 °C, (c) 900 °C, (d) 1000 °C; Figure S5: Room temperature frequency dependent C-V, G-V characteristics and corresponding conductance peak position and width for Ge NCs with different growth temperature (a–c) 800 °C and (d–f) 900 °C.
