# Supplementary material for: Tailoring Ge Nanocrystals via Ag-Catalyzed Chemical Vapor Deposition to Enhance the Performance of Non-Volatile Memory
Source: Nanomaterials (Basel). 2026 Jan 22;16(2):146. doi: 10.3390/nano16020146 (PMC12844894; doi:10.3390/nano16020146)
Supplement: Supplementary file 1 [file nanomaterials-16-00146-s001.zip › nanomaterials-4055871-supplementary.pdf]

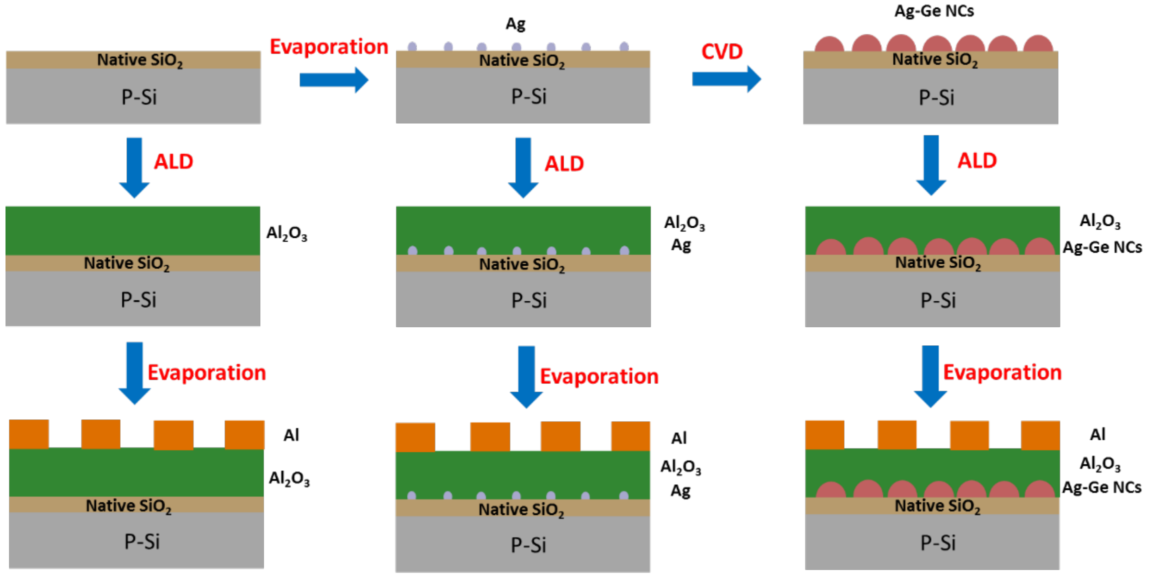

Figure S1. Preparation flowchart of control samples (p-Si/SiO_2/Al_2O_3/Al and p-Si/SiO_2/Ag/Al_2O_3/Al).

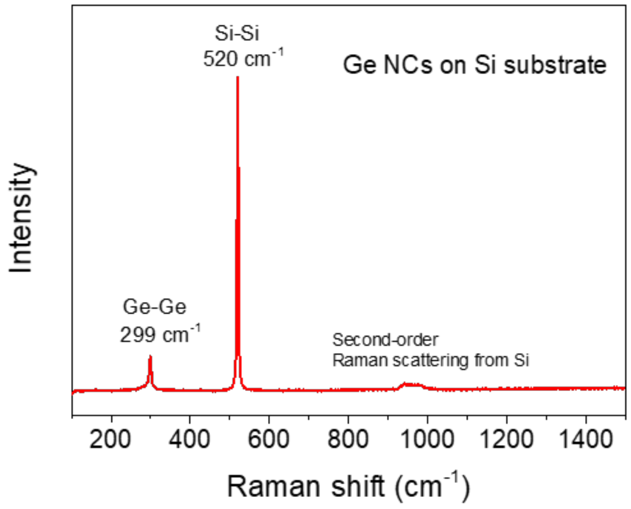

Figure S2. The Raman spectrum of the synthesized Ge NCs at 700 °C.

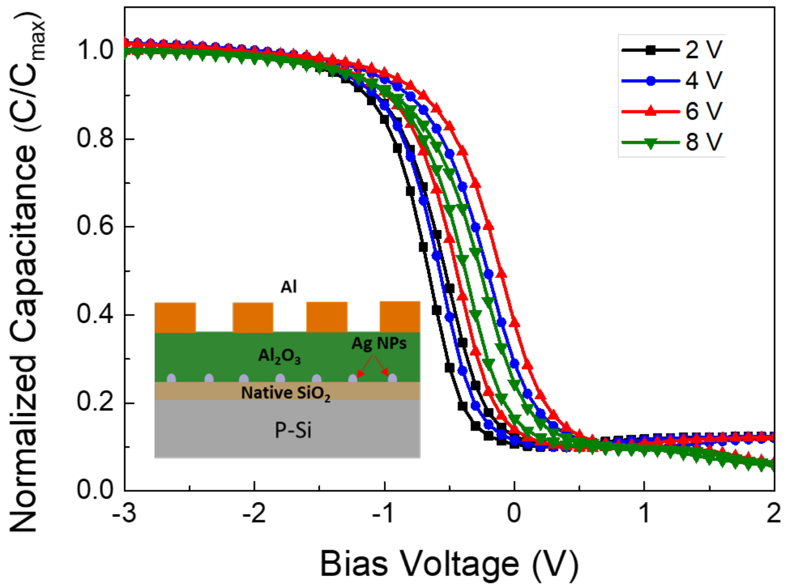

Figure S3. High frequency (1 MHz) C-V hysteresis behavior of the control sample (p-Si/SiO_2/Ag NPs/Al_2O_3/Al).

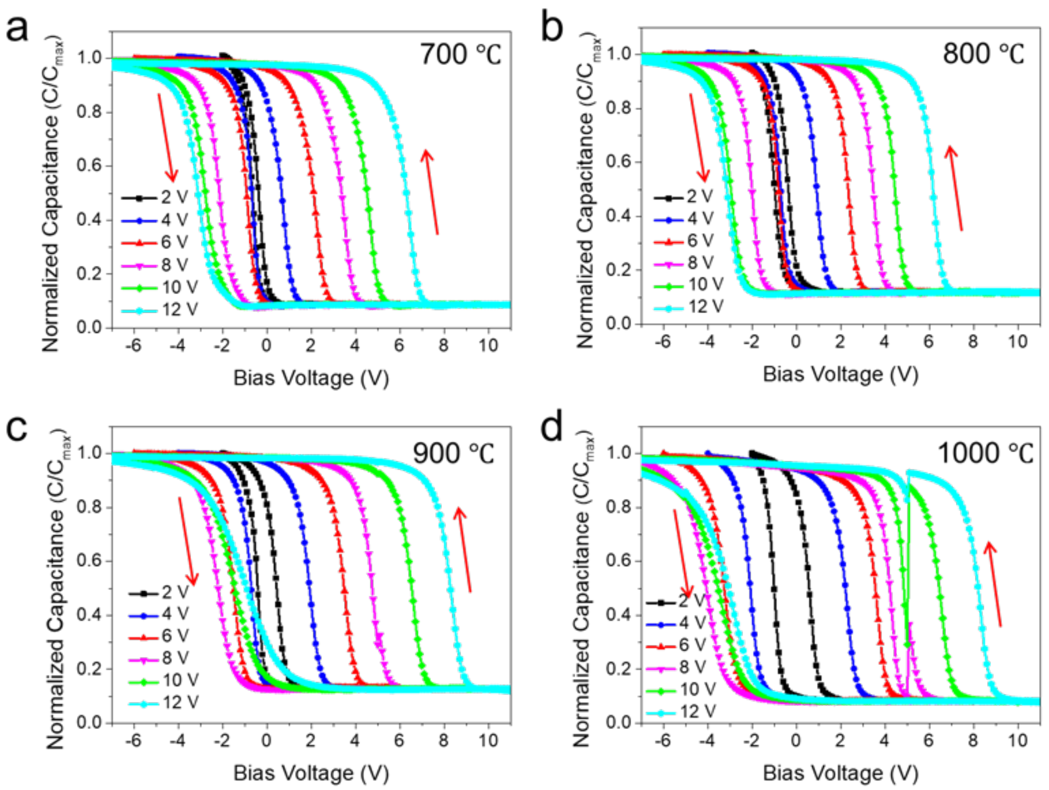

Figure S4. High frequency (1 MHz) C-V hysteresis behavior of (e) the control sample and the MIS structures of Ge NCs with different growth temperature (a) 700 °C, (b) 800 °C, (c) 900 °C, (d) 1000 °C.

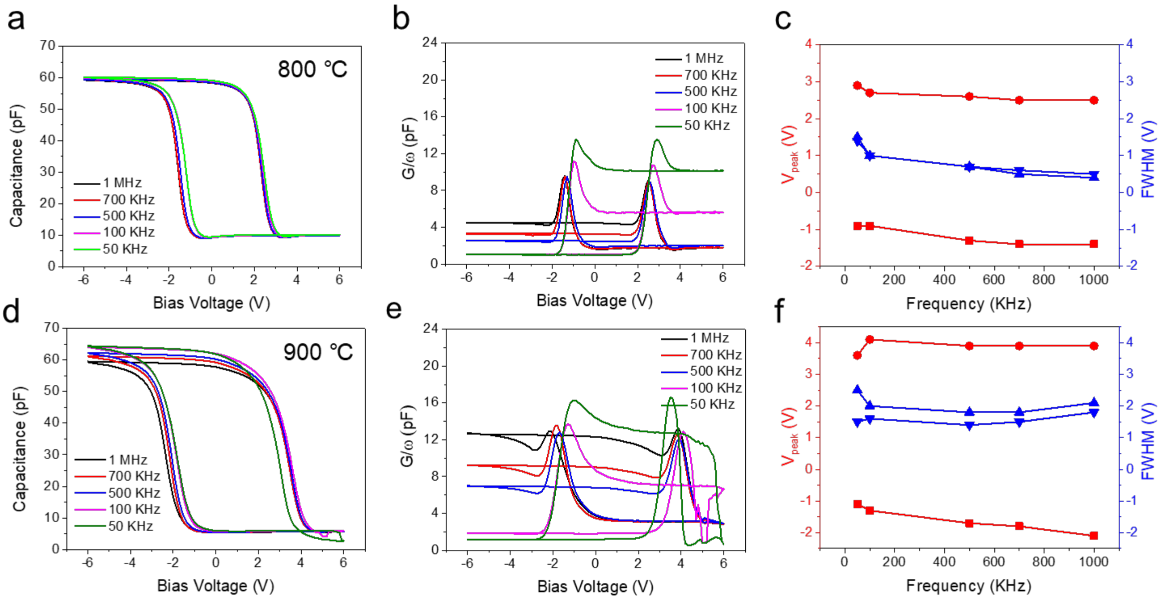

Figure S5. Room temperature frequency dependent C-V, G-V characteristics and corresponding conductance peak position and width for Ge NCs with different growth temperature (a, b, c) 800 °C and (d, e, f) 900 °C.
